# ARTEM server: an online tool for nucleic acid 3D motif searches, 3D structure superposition and structure-based alignment

**DOI:** 10.1093/nar/gkag428

**Published:** 2026-05-05

**Authors:** Dominik Sordyl, Jan Cielesz, Davyd R Bohdan, Eugene F Baulin, Janusz M Bujnicki

**Affiliations:** Laboratory of Bioinformatics and Protein Engineering, International Institute of Molecular and Cell Biology in Warsaw, ul. Ks. Trojdena 4, PL-02-109 Warsaw, Poland; Laboratory of Bioinformatics and Protein Engineering, International Institute of Molecular and Cell Biology in Warsaw, ul. Ks. Trojdena 4, PL-02-109 Warsaw, Poland; Laboratory of Bioinformatics and Protein Engineering, International Institute of Molecular and Cell Biology in Warsaw, ul. Ks. Trojdena 4, PL-02-109 Warsaw, Poland; Laboratory of RNA Algorithms, IMol Polish Academy of Sciences, ul. M. Flisa 6, PL-02-247 Warsaw, Poland; Laboratory of Bioinformatics and Protein Engineering, International Institute of Molecular and Cell Biology in Warsaw, ul. Ks. Trojdena 4, PL-02-109 Warsaw, Poland; Laboratory of Bioinformatics and Protein Engineering, International Institute of Molecular and Cell Biology in Warsaw, ul. Ks. Trojdena 4, PL-02-109 Warsaw, Poland

## Abstract

ARTEM Server is an online platform for comparative analysis of nucleic acid 3D structures, combining two complementary superposition methods based on the ARTEM algorithm. The server provides access to searches for local tertiary motifs using the ARTEM tool, which identifies local isosteric structural arrangements without relying on sequence, interaction annotations, or backbone connectivity. It also offers global structure alignment and search modes via ARTEMIS, a recent extension of ARTEM that performs global sequence alignments based on rigid-body structural superposition. ARTEMIS supports both classical sequentially ordered superpositions and alignments involving sequence permutations, and can enumerate alternative suboptimal matches, enabling structural searches within large molecules or across databases. Benchmarks reported in the original publications demonstrate that ARTEM and ARTEMIS outperform other tools and are particularly effective at detecting 3D motif and 3D fold similarities across diverse backbone contexts, including cases that are challenging for sequence-ordered or annotation-dependent methods. ARTEM Server unifies these capabilities in a web interface, accepting PDB/mmCIF inputs, supporting multiple query and reference structures, and providing interactive 3D visualization and exportable alignment and motif-matching data. ARTEM Server offers a user-friendly web-based environment for exploration of global nucleic acid folds and local tertiary motifs. The web server is available at https://artemserver.genesilico.pl/.

## Graphical Abstract

**Graphical Abstract:**
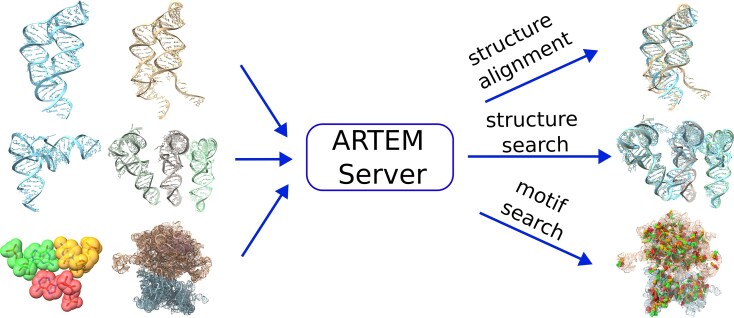

## Introduction

RNA three-dimensional (3D) structures are central to understanding the functions of noncoding RNAs, which often depend more on tertiary structure than on primary sequence conservation. The RNA structural landscape is highly modular and can be decomposed into recurrent tertiary motifs of various sizes, spanning local motifs embedded within secondary structure elements as well as long-range contacts. Multiple motif types, such as G-quadruplexes and GNRA tetraloops, occur in both RNA and DNA structures [1]. This has motivated the development of a computational methods package that compares nucleic acid structures directly at the level of 3D coordinates rather than relying solely on sequence or secondary structure.

Two broad classes of nucleic acid 3D structure comparison tools have emerged, mostly applied to RNA. The first focuses on global pairwise alignment of whole RNA molecules to derive structure-based sequence alignments, quantify fold similarity, and analyze conformational changes. Early approaches such as DIAL [2], ARTS [3], R3D Align [4], STAR3D [5], and SupeRNAlign [6] combine rigid-body superpositions at the global-only or global and local levels with variants of dynamic programming to achieve flexible sequence alignments. Later methods, including RMalign [7], RNA-align [8], and related tools, further refined scoring functions and heuristics to improve alignment quality and runtime on large RNAs. More recently, US-align was introduced as a general framework for universal structure alignment of proteins, nucleic acids and complexes, using Template Modeling (TM)-score-like metrics to balance coverage and geometric similarity [9]. However, most of these methods assume a sequentially ordered alignment, in which residue indices in both chains are required to be monotonically increasing. This assumption is appropriate for homologous RNAs with conserved topology, but it breaks down in many biologically relevant cases, such as circular or noncircular permutations as found in transfer RNA (tRNA)-like viral RNAs [10], hammerhead ribozyme variants [11], or in complex motifs such as G-quadruplexes, which are known to adopt multiple backbone topologies [12].

The second class of methods targets local structural motifs rather than entire molecules. Template-based motif scanners such as NASSAM [13], FR3D [14], and RNAMotifScanX [15] search for instances of known tertiary motifs (e.g. GNRA tetraloops [16], ribose zippers [17], loop-receptor contacts [18]) across structural databases, typically operating at the level of annotated base pairs, stacking, and other interactions. More recent tools, including LocalSTAR3D [19], CircularSTAR3D [20], and RNAMotifContrast [21], emphasize local stack-based representations or graph alignments to detect motif subfamilies and circular permutations, while remaining tied to specific interaction or sequence patterns. These approaches have enabled detailed cataloguing of local motifs in curated datasets, but they are inherently constrained by motif definitions that depend on sequence signatures, pre-annotated base-pairing networks, and explicit secondary structure contexts.

Several web servers have sought to make nucleic acid 3D comparison more accessible by wrapping global or local alignment methods in interactive interfaces. SETTER provides an RNA structure comparison server based on secondary structure elements, decomposing molecules into stems and loops and aligning these building blocks [22]. Rclick offers a topology-independent comparison of RNA 3D structures but is optimized for producing sequence alignments, assuming a particular decomposition and relying on specific geometric descriptors [23]. While these servers are widely used and effective within their intended scopes, their dependence on secondary structure segmentation or on sequential alignment schemes can limit their ability to detect structurally isosteric motifs embedded in different backbone topologies or to explore long-range motifs that span multiple chains and pseudoknotted regions.

The limitations of both global and local approaches are particularly acute for long-range tertiary motifs and for motifs that recur in different backbone topologies. Even the most flexible motif catalogues are typically built on interaction annotations produced by tools such as DSSR [24], FR3D [14], MC-Annotate [25], RNAView [26], or ClaRNA [27], which can disagree on noncanonical base pairs and higher-order contacts [28]. Motif definitions based on secondary structure elements also enforce a fixed loop or junction type, making it difficult to recognize that a given tertiary architecture can occur as an internal loop, a multiway junction, or a long-range helix–helix interface. At the same time, global alignment tools treat the molecule as a single chain and are not naturally suited to searching large structure databases for local, recurring 3D arrangements. Finally, the RNA 3D alignment problem is computationally challenging: exhaustive topology-independent alignment has exponential complexity, and current methods rely either on very simple, fast heuristics or on intricate but slow strategies that do not scale well to large searches [29].

The ARTEM family of methods was developed to address these gaps by operating directly on 3D coordinate representations of nucleic acids without assuming any particular sequence, interaction, or topology model. ARTEM, in its original formulation, provides annotation-, sequence-, and topology-independent superposition of arbitrary RNA 3D modules and was used to construct the LORA dataset of long-range RNA modules and to identify common long-range tertiary motifs [28]. ARTEM 2.0 extends this concept into a general tertiary motif search engine for RNA and DNA that uses superposition of base arrangements to detect local motifs across all backbone contexts, including long-range modules, without relying on secondary structure type or base-pair annotations [1]. Building on the same core idea, ARTEMIS introduces a polynomial-time heuristic for global rigid-body alignment that unifies sequentially ordered and topology-independent superpositions within a single framework and produces structure-based sequence alignments scored by TM-Score_RNA_ [29]. Together, ARTEM and ARTEMIS enable unbiased comparison of both local motifs and global folds across arbitrary backbone topologies. ARTEM Server integrates these complementary capabilities in a web-accessible environment, allowing users to perform topology-independent motif searches and global structure comparisons on nucleic acid 3D structures and databases within a single, unified workflow.

## Materials and methods

ARTEM Server is implemented using React [30] for the front end and Django [31] for the back end. It supports all modern web browsers, and operates on a dedicated virtual machine hosted by the International Institute of Molecular and Cell Biology in Warsaw (IIMCB). ARTEM Server provides three computational workflows: global structure alignment, structure search, and motif search. All workflows begin with structure acquisition. Users may upload coordinate files in PDB or mmCIF format with a maximum size of 15 MB or specify PDB identifiers to retrieve structures directly from the Protein Data Bank via the PDB API. Processing of the input files and optional user-specified chain or residue ranges is handled directly by the original ARTEM and ARTEMIS tools. Each workflow is managed by an independent job queue implemented in Celery [32]; each job is assigned a unique identifier and stored for 30 days. 3D visualizations are implemented using the Mol* plugin [33]. For database searches, the BGSU representative set of RNA structures [34] is stored on the server and updated monthly. All workflows conclude with automated generation of visualization-ready files and tabular reports. The server prepares Mol* sessions with preloaded superposed structures or matched motifs, and provides downloadable coordinate files, alignment files, residue-index mappings, and metadata describing all parameters used in the computation.

In the global alignment workflow, up to 10 reference structures are compared against up to 10 query structures by running pairwise alignments with ARTEMIS [29]. After validation, the server computes all residue–residue seed superpositions, or a reduced seed set when the smaller structure exceeds 500 nts. For each seed, ARTEMIS identifies mutually closest residues under a strict matching threshold and derives candidate matchings. Both topology-independent and sequentially ordered matchings are generated, and ARTEMIS refines each candidate by repeating rigid-body fitting, selecting the superposition that maximizes the TM-score_RNA_. For each reference-query pair, the server stores the superimposed query coordinates, the inferred residue correspondence, Root-Mean-Square Deviation (RMSD), TM-score_RNA_ values, coverage values, and the structure-based sequence alignment. Format conversion mode is enabled when a single query structure is provided without a reference, with no file size limit applied.

In the structure search workflow, ARTEMIS is executed in repeated-alignment mode, with optional reporting of alternative suboptimal matches. The user provides a single query structure and either a set of up to 10 reference structures or selects the database search option. In database mode, the server screens the query structure against the BGSU representative set of RNA structures. All superpositions that satisfy the user-defined TM-score_RNA_ threshold are retained. For a set of reference structures, the output includes a ranked list of all matches, downloadable structural and tabular reports, and a 3D visualization frame, whereas in the database search mode, only the ranked list of all matches is available.

In the motif search workflow, the ARTEM tool [1] is used to identify all local superpositions of the query motif, up to 25 residues in size, within each structure of the input reference set or the database. The server extracts the nucleotide fragment defined by the user as the query motif and generates residue-residue seed superpositions across all possible positions in the reference structure. For each seed, ARTEM identifies mutually closest residues using its local geometric matching threshold and returns all matches whose size and RMSD satisfy the user-defined constraints. As in the structure search workflow, for a set of reference structures, the output includes a ranked list of all matches, downloadable structural and tabular reports, and a 3D visualization frame, whereas in the database search mode, only the ranked list of all matches is available. The database search mode reports up to 1,000 identified matches.

The three workflows implemented in ARTEM Server address different comparison scales. The global alignment workflow is intended for whole-structure comparisons and generation of structure-based sequence alignments, including cases involving circular or noncircular permutations. The structure search workflow is suitable for identifying occurrences of a larger structural fragment within reference structures or databases, particularly when multiple or partial matches are expected. The motif search workflow is optimized for compact tertiary motifs, typically up to 25 residues, and is most appropriate when a specific residue arrangement is used as a query. Selecting the workflow that matches the intended scope of the analysis generally improves interpretability and computational efficiency.

## Results

### Example applications

#### Structure alignment

**Figure 1.**
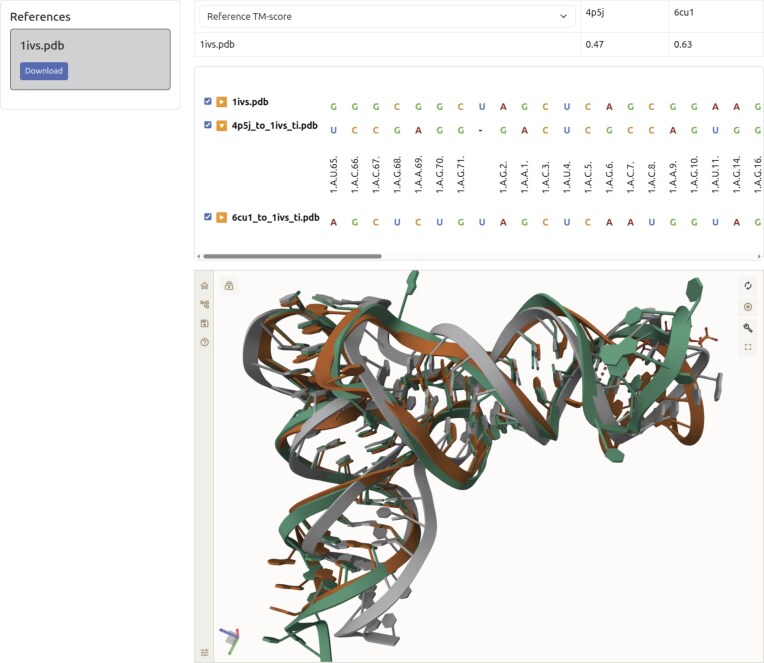
Demonstration of the ARTEM Server global structure alignment workflow output page (https://artemserver.genesilico.pl/result/exmpA2/align). A reference tRNA structure (PDB entry 1IVS) is compared with a viral tRNA-like molecule (PDB entry 4P5J) and a bacterial Y RNA (PDB entry 6CU1). In the Mol* frame, tRNA chain C, used for the superposition, is shown in green, the viral tRNA-like structure in white, and the Y RNA in red. The residue identifier section within the alignment view is expanded for the viral tRNA-like molecule and demonstrates the permuted matching to the tRNA.

Figure 1 illustrates the comparison of two tRNA-like molecules with a tRNA structure (https://artemserver.genesilico.pl/result/exmpA2/align). A tRNA structure (PDB entry 1IVS) [35] was selected as the reference, and a viral tRNA-like structure (PDB entry 4P5J) [36] and a bacterial Y RNA (PDB entry 6CU1) [37] were selected as queries. Because the tRNA in 1IVS is represented by two copies, only chain C was specified in the “Select reference residues to consider” field of the Advanced Options on the input page. The results page shows a reference TM-score of 0.47 for the viral tRNA-like structure (4P5J) and 0.63 for the Y RNA (6CU1). The sequence alignment features files “4p5j_to_1ivs_ti.pdb” and “6cu1_to_1ivs_ti.pdb”, where “ti” stands for topology-independent, indicating that the optimal superpositions identified by ARTEMIS include sequence permutations. Thus, ARTEMIS successfully identified the circularly permuted similarity between 1IVS and 6CU1, as well as the noncircularly permuted similarity between 1IVS and 4P5J. This can be verified by expanding the residue identifiers. The 3D viewer displays the tRNA chain C from 1IVS, along with the two superimposed query structures.

We repeated the same comparison using similar web servers. The US-align server (https://aideepmed.com/US-align/) [9] correctly aligned the viral tRNA-like structure, although with a slightly lower reference TM-score of 0.42, and failed to align the bacterial Y RNA (reference TM-score of 0.36). Notably, a locally installed US-align (version 20 220 227) successfully aligned the same Y RNA in our previous benchmark, see table 1 in [29]. The Rclick server (https://mspc.bii.a-star.edu.sg/minhn/rclick.html) [23] reported alignments similar to those obtained with the ARTEM Server, but with lower reference coverage (0.65 versus 0.84 for 4P5J and 0.79 versus 0.91 for 6CU1). Similarly, the RNAhugs server (https://rnahugs.cs.put.poznan.pl/) [38] reported the correct superpositions but with lower reference coverage (0.76 versus 0.84 for 4P5J and 0.51 versus 0.91 for 6CU1). Both Rclick and RNAhugs do not report TM-score values. Furthermore, US-align and Rclick accept only one reference structure and one query structure at a time. Taken together, these comparisons illustrate that the ARTEM Server provides complementary capabilities and, in the tested cases, achieves higher coverage and improved alignments compared to the other methods.

#### Structure search

Here, we screened the helical-packing motif structure against two riboswitch molecules (https://artemserver.genesilico.pl/result/exmpS2/search). The helical-packing motif [29] from a THF riboswitch (PDB entry 6Q57) was selected as a query to screen against two references: M-box RNA (PDB entry 3PDR) [39] and Lysine riboswitch (PDB entry 3DIG) [40]. Because the references may contain multiple instances of the motif, the structure alignment workflow is not applicable here. Moreover, the motif is relatively large (50 residues) and is expected to generate multiple partial hits matching individual stems, which justifies the use of the structure search workflow with a global TM-score-based threshold rather than the motif search workflow, which is more suitable for smaller motifs. The TM-score threshold was set to 0.4, and the “Show sub-optimal matches” option was enabled. The results page shows two motif matches for 3PDR with TM-scores of 0.46 and 0.45, and one match of TM-score 0.5 for 3DIG. As in the other workflows, the left panel allows the user to choose the reference structure, whose matches will appear in the sequence alignment segment and the 3D viewer, by clicking on its name. In both reference tabs, the query motif’s sequence is labeled as “#0″ in the sequence alignment segment. To the best of our knowledge, no web server with comparable structure search functionality is available.

#### Motif search

Here, we screened the kink-turn motif against a group II intron (https://artemserver.genesilico.pl/result/exmpM1/motifsearch). An instance of the kink-turn 7 motif from PDB entry 1FFK [41] was selected as the query structure and searched against a group II intron reference structure from PDB entry 7UIN [42]. The minimum match size was set to 11 residues, with no RMSD thresholds. The results page lists six unique kink-turn matches: two of size 12 and four of size 11. The query motif’s sequence is labeled as “#0 kt7_13res_template.pdb” in the sequence alignment segment. The 3D viewer shows that four of the six matches heavily overlap, indicating that the six matches correspond to three distinct motif instances in the 7UIN structure. Visual inspection confirms that two of the instances belong to the canonical internal loop variant of the kink-turn, whereas the third instance (labeled as “#2 RMSD: 2.2″) represents a no-kink variant of the motif [1]. For comparison, DSSR (version 2.0) [24] identified a single kink-turn in the 7UIN structure, annotated as “Normal k-turn” (labeled as “#4 RMSD: 1.55″), and reported a false positive hit annotated as “Undecided case”. Thus, the ARTEM Server combines superior performance with the advantage of being accessible via the web.

In another example (https://artemserver.genesilico.pl/result/exmpM4/motifsearch), we screened a G-tetrad instance from PDB entry 2RQJ [43] against three G-quadruplex variants: a two-strand DNA variant (1A8W [44]), a four-strand DNA-RNA hybrid variant (6FFR [45]), and a single-strand RNA G-quadruplex (4XK0 [46]). The maximum match RMSD was set to 1.0 Å, and the minimum match size was set to 4. ARTEM identified eight out of nine tetrads across the three G-quadruplexes. The only absent tetrad was a flanking RNA tetrad, which, due to a slightly distorted conformation, could be identified only as a three-residue match with default ARTEM settings. This example highlights the applicability of ARTEM to RNA, DNA, and hybrid nucleic acid structures.

## Discussion

Existing approaches to RNA and DNA 3D structure comparison tend to separate global alignment from motif detection and rely on assumptions about sequence order, backbone connectivity, or pre-annotated interaction networks. As a consequence, current tools struggle to recognize relationships between structures that differ in topology or contain recurrent tertiary motifs embedded in distinct secondary structure contexts. These observations highlight the need for methods capable of operating directly on the geometric information encoded in 3D coordinates, particularly in cases where sequence order or secondary structure annotations are insufficient to capture underlying structural relationships.

The ARTEM algorithm was developed in response to these limitations. Its two branches: (i) ARTEM for local motif searches and (ii) ARTEMIS for global alignments, provide complementary solutions. ARTEM Server integrates these capabilities into a unified, web-accessible system, enabling searches for both global structural similarity and local isosteric motifs within a single computational environment. By supporting topology-independent alignments, sequence permutations, and unconstrained motif searches, the server is well suited for exploring RNA and DNA structures beyond typical arrangements of helices, loops, and junctions, or structures whose motifs recur in unexpected architectural settings. The ability to perform structure searches against user-defined collections or curated databases further extends its applicability to comparative genomics, structural classification and the discovery of motif variants.

The workflows implemented in ARTEM Server allow users to examine relationships between nucleic acid structures at different spatial scales, from whole-molecule superpositions to compact tertiary motifs, using a consistent geometric framework. This integration enables analyses that were previously difficult to perform across disparate tools and provides a platform for continued refinement as new RNA 3D folds and motifs continue to be discovered.

Some limitations remain for future improvements. ARTEM Server relies on rigid-body superposition and does not explicitly account for conformational flexibility, which can affect performance for highly dynamic regions or low-resolution structures. Motif searches are restricted to relatively compact fragments, and topology-independent matching may produce alternative superpositions that require expert interpretation to assess biological relevance. Addressing these limitations, including improved handling of structural variability, larger and more diffuse motifs, and increased robustness to lower-quality input models, will be among the targets of future development of the ARTEM methods and subsequent versions of the web server.

## Data Availability

The web server is available at https://artemserver.genesilico.pl/. This website is free and open to all users and there is no login requirement.
